# Posterior Inferior Cerebellar Artery Infarct Associated With Cervical Dystonia: A Case Report

**DOI:** 10.7759/cureus.90682

**Published:** 2025-08-21

**Authors:** Anandu M Anto, Saran Lal A Mokan Dasan, Marcos Molina, Navya Mandalapu, Manjeet Dhallu

**Affiliations:** 1 Internal Medicine, BronxCare Health System, New York City, USA; 2 Neurology, BronxCare Health System, New York City, USA

**Keywords:** acute ischemic stroke (ais), cerebro-vascular accident (stroke), cervical dystonia, posterior inferior cerebellar syndrome, secondary dystonia

## Abstract

Cerebellar infarcts, particularly those involving the posterior inferior cerebellar artery (PICA), can present with diverse and atypical neurological symptoms that complicate timely diagnosis. We present the case of an 84-year-old woman with a history of vascular risk factors who developed sudden-onset dizziness, vomiting, and altered mental status, ultimately found to have a right PICA territory infarct on MRI. Notably, on the second day of admission, she developed transient cervical dystonia (CD) characterized by leftward neck deviation and sternocleidomastoid spasm. This focal motor manifestation, in the absence of classic ataxia or cranial nerve deficits, highlights the rare but significant association between cerebellar stroke and dystonia.

Neuroanatomical pathways involving the cerebellum, basal ganglia, and vestibular systems, particularly the Guillain-Mollaret triangle (GMT) and corticocerebellar loops, may underlie such movement disorders. Additionally, ischemia of structures such as the accessory nerve or imbalance in vestibular tone may contribute to cervical posturing. The patient’s dystonia improved spontaneously with supportive care and baclofen, supporting the theory of transient post-ischemic hypersensitivity rather than fixed structural damage.

This case emphasizes the importance of recognizing central causes of acquired torticollis to avoid misdiagnosis and delays in stroke management, particularly in elderly patients presenting with subtle posterior circulation symptoms.

## Introduction

Cerebellar strokes, particularly those involving the posterior inferior cerebellar artery (PICA), are recognized for their diverse and often atypical presentations, a phenomenon increasingly identified since the widespread use of computed tomography in the 1980s [1]. Cerebellar infarcts account for approximately 2% (range 1.5-2.3%) of all cerebral infarctions, with PICA strokes representing roughly 40% of cerebellar ischemic events [1-4]. While classic symptoms include vertigo, ataxia, and cranial nerve deficits, less common manifestations such as focal dystonia have been described [5]. Early diagnosis can be challenging due to subtle initial symptoms and the limited sensitivity of CT imaging for posterior circulation infarcts [3]. Though rare, isolated case reports have documented cervical dystonia (CD) appearing from days to months after posterior circulation strokes [6-8]. The cortex, basal ganglia, and cerebellum each represent a node in the motor pathway, and dysfunction in any of these nodes can cause dystonia [9,10]. While dystonia has been noted in the chronic stages of stroke, acute dystonia as a symptom of acute stroke remains a clinical oddity. Yoram RC et al., in their literature review of nine cases of acute dystonia following an acute ischemic stroke, reported only one cerebellar stroke preceding acute dystonia [11]. Although post-stroke movement disorders can be hypokinetic or hyperkinetic, hyperkinetic movement disorders, of which dystonia is a type, are the most common clinical finding [10,11]. We present a case of cerebellar stroke that initially manifested as vertebrobasilar insufficiency and subsequently developed acute transient CD. This sequence of symptoms is clinically relevant because vertebrobasilar insufficiency may mimic benign vestibular syndromes, potentially delaying recognition of posterior circulation strokes. The subsequent emergence of dystonia, particularly in the acute phase, may further confound diagnosis, underscoring the need for heightened clinical suspicion when atypical motor findings accompany posterior circulation symptoms.

## Case presentation

An 84-year-old woman with a history of hypertension, hyperlipidemia, and cerebrovascular accident with residual left-sided facial palsy presented to the ED in February 2025 with sudden-onset dizziness, headache, nausea, vomiting, and altered mental status. On arrival, her blood pressure was 181/75 mmHg, and her heart rate was irregularly irregular at 75 beats per minute. General examination was otherwise unremarkable. Neurologically, she was alert but oriented only to person. Cranial nerve examination revealed residual upper motor neuron facial palsy, motor power 5/5 bilaterally, and intact sensation in all limbs with no pathological reflexes. The patient did not have palatal deviation, a deviated uvula, or nasal regurgitation of food. She was noted to have right-sided past pointing and abnormal dysdiadochokinesis. Laboratory investigations on admission were unremarkable.

**Figure 1:**
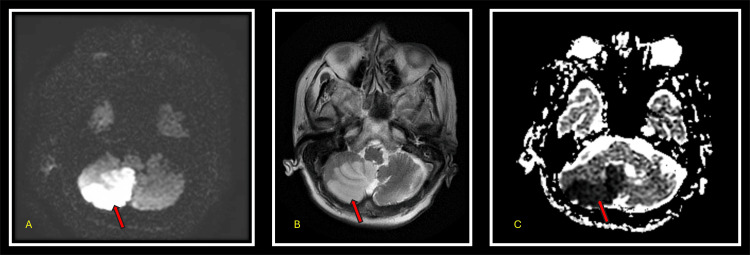
MRI brain showing right-sided ischemic cerebellar stroke. The red arrow points to the same lesion across different MRI sequences, the combination of which supports the diagnosis of an acute infarct. The lesion appears hyperintense on axial diffusion sequence (A) and axial T2 (B), and hypointense on apparent diffusion coefficient (C), consistent with diffusion restriction suggestive of acute infarct.

A non-contrast CT scan of the head performed in the ED showed no acute pathology. However, an MRI of the brain performed on the second day of admission demonstrated an acute, diffusion-restricted infarct in the posterior inferior right cerebellar hemisphere, along with evidence of an old infarct and encephalomalacia in the right frontal operculum and insula (Figure 1).

The patient was started on dual antiplatelet therapy (DAPT) as her NIHSS score was 2. On day 2 of admission, she developed deviation of the neck to the left with inability to move her neck to the right. The left sternocleidomastoid (SCM) appeared tight and in spasm. Neurology was consulted, and she was diagnosed with acute cerebellar stroke with CD. Telemetry revealed new-onset atrial fibrillation with a controlled ventricular rate. As per Neurology recommendations, DAPT was discontinued and apixaban was started. She was continued on apixaban and a statin, and baclofen was initiated for dystonia. At her one-week follow-up after discharge, her dystonic symptoms had significantly improved, and she was able to walk independently without dizziness.

## Discussion

Cervical dystonia (CD) is the most common form of focal dystonia, with an incidence of 1.18 per 100,000 person-years [12]. While most cases are idiopathic, secondary causes include perinatal insults, vascular events, trauma, infections, and drug-induced processes affecting the central and peripheral nervous systems. Dystonia is a complex motor disorder involving the motor cortex, basal ganglia, and cerebellum, regions that collaboratively refine motor signals to ensure coordinated movement. The incidence of CD after a cerebellopontine stroke is so infrequent that a systematic review by Ogawa T et al. in 2018 found only a handful of such cases. They identified a total of 18 cases of secondary CD due to brainstem lesions between 1979 and 2016 [8]. The age of affected patients ranged from 15 months to 86 years, with a heterogeneous phenotype of dystonia, 25% of which were ipsilateral to the side of the lesion. This review also noted that a single brainstem lesion was sufficient to induce CD, as multiple brainstem lesions were reported in only 4 of the 18 patients [8].

The PICA has the most complex relationships with the cranial nerves as it courses close to the glossopharyngeal, vagus, accessory, and hypoglossal nerves [13]. This proximity between the PICA and the accessory nerve, which innervates the sternocleidomastoid muscle, could explain sternocleidomastoid spasm through accessory nerve dysfunction during ischemic injury. Neuroanatomically, the cerebellum is divided into three functional regions: the vestibulocerebellum (balance and eye movements), spinocerebellum (trunk and limb coordination), and cerebrocerebellum (planning and modulation of voluntary movements). The PICA’s vascular territory overlaps with several critical cerebellar and medullary structures, and infarction in this region can therefore lead to a wide range of clinical syndromes, from classic lateral medullary syndrome to isolated cerebellar symptoms or, as in our case, focal dystonia without ataxia. The Guillain-Mollaret triangle (GMT), also known as the dentato-rubro-olivary circuit or myoclonic triangle, and the corticocerebellar loops are the two main feedback loops responsible for active movements, motor accuracy, and coordination [14]. The dentato-rubro-olivary circuit connects the dentate nucleus in the cerebellum with the contralateral red nucleus and inferior olivary nucleus in the brainstem via the superior cerebellar peduncle, central tegmental tract, and inferior cerebellar peduncle [15]. The corticocerebellar loop connects the motor cortex to the cerebellar cortex (via pontine nuclei), which in turn projects primarily to the VL nuclei of the thalamus and then back to the cerebral cortex [10]. The GMT, which forms a functional neuronal connection between the cerebellum and basal ganglia, of which the inferior olivary nucleus is a part, has been noted to be hypertrophied in patients with stroke and chronic dystonia [15,16]. The loss of inhibitory signals from the dentate nucleus may be the cause of this hypertrophy [15]. Although a causative role cannot be established due to temporal dispersion of the findings, this provides a possible link between dystonia and the GMT.

**Figure 2:**
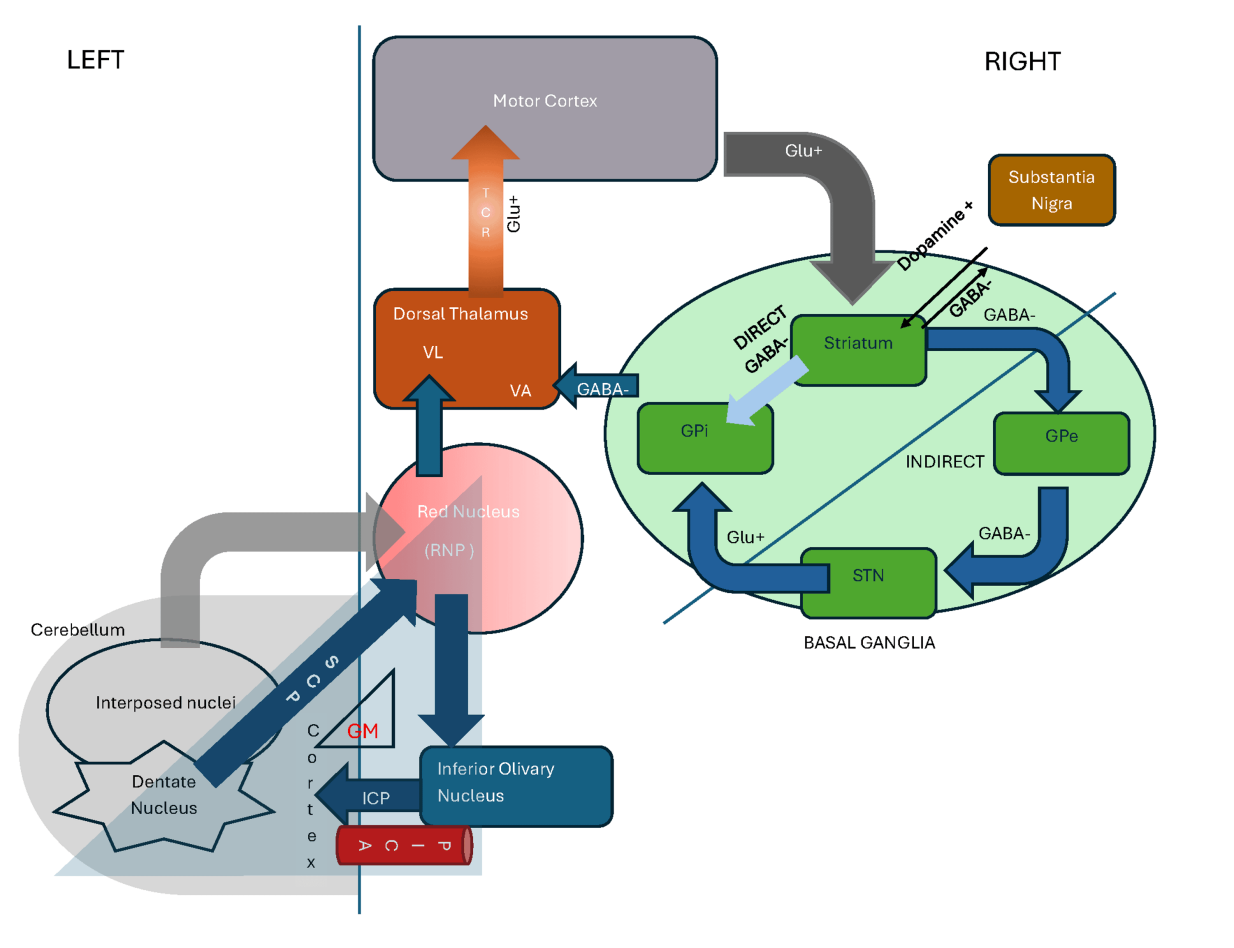
Author-proposed schematic diagram illustrating the neural pathways of motor coordination, highlighting interactions between the cerebellum and basal ganglia. This model reflects the authors’ interpretation of current evidence. VL: Ventral Lateral; VA: Ventral Anterior; GPi: Globus Pallidus Internus; GPe: Globus Pallidus Externus; STN: Subthalamic Nucleus; SCP: Superior Cerebellar Peduncle; ICP: Inferior Cerebellar Peduncle; RNP: Parvocellular Red Nucleus; GM: Guillain–Mollaret Triangle; PICA: Posterior Inferior Cerebellar Artery; GABA: Gamma-Aminobutyric Acid; Glu: Glutamate

The basal ganglia control of movement occurs via the direct and indirect pathways. Normally, there is a balance between inhibitory and excitatory influences. The direct pathway is excitatory, resulting from the disinhibition of upper motor neurons via connections from the caudate and putamen to the internal segment of the globus pallidus and the substantia nigra [11]. In contrast, the indirect pathway modulates (i.e., counterbalances) the direct pathway; activation of the indirect pathway leads to inhibition of the upper motor neurons [11]. Depending on the net inhibition of the upper motor neuron (UMNs), the corticospinal output is adjusted, leading to varying movement amplitudes (Figure 2).

Cerebellar control of movement passes through the ventrolateral (VL) nuclei of the dorsal thalamus. The basal ganglia, in turn, stimulate the ventroanterior (VA) nuclei of the dorsal thalamus. The combined output then projects to the motor cortex via thalamocortical radiations. Interruption of connections within the basal ganglia, particularly to the putamen, striatum, and pallidum, can increase thalamic drive to frontal and motor cortical areas, resulting in dystonia or other movement disorders such as tremor, myoclonus, ballism, chorea, and athetosis [17,18].

Although no concrete neuropathological or electrophysiological evidence exists regarding the pathogenesis of stroke-related dyskinesia, many hypotheses have been postulated. One such hypothesis is post-synaptic denervation hypersensitivity, along with impaired plasticity of axons and dendrites after ischemic injury [10]. There is no single motor pathway explanation for dystonia, but rather a multimodal disruption that could explain the occurrence of dystonia in diverse clinical settings [19]. Liuzzi D et al. reported that upper limb dystonia was more frequently associated with lesions in the thalamus (39 cases) and basal ganglia (17 cases), compared to lesions in the brainstem (4 cases) and cerebellum (1 case) [19,20]. LeDoux MS and Brady KA found that among 25 cases of CD, only 7 had lesions located in the basal ganglia, while the rest were in the cerebellum, brainstem, and spinal cord, similar to other individual case reports [7,19,21,22]. All these findings point towards a multimodal rather than a single anatomical disruption.

Our patient with a PICA ischemic stroke initially presented with cerebellar signs such as nausea, vomiting, dizziness, and a change in baseline mental status, and subsequently developed a secondary dystonic reaction. Considering the above-discussed anatomy and the patient’s MRI, which showed a PICA stroke, we hypothesize that the ischemia might have caused disruption in the cerebellar node of the motor pathway, leading to interruption of motor feedback. This temporary disruption due to ischemia led to hypersensitivity of the afferent motor pathway and, consequently, a dystonic reaction, which eventually improved by itself without active intervention [10].

Another perspective in this case is that in patients with PICA stroke but milder symptoms, a presentation such as neck tilt can be mistaken for posturing secondary to vestibular disorders or torticollis. In a series of five patients ultimately diagnosed with PICA infarcts, all were initially evaluated for vertigo and underwent peripheral vestibular testing before stroke was recognized or suspected [23]. Recognizing stroke as a potential cause of acquired torticollis is critical, as misdiagnosis can delay time-sensitive interventions. Several non-stroke conditions can mimic this presentation (Table 1).

**Table 1:** Selected case reports of non-stroke causes of neck tilt.

Study	Age/Sex	Diagnosis	Key Features
Oyama H et al. [24]	66M	Posterior fossa meningioma	5-week history of torticollis, ataxia
Burke RE et al. [25]	52F	Tardive dystonia	Chronic metoclopramide use
Greenberg MR et al. [26]	38F	Atlantoaxial rotatory subluxation	Torticollis, cervical tenderness; no focal neurologic deficits
Filip P et al. [27]	61F	Vestibular schwannoma	2-month history of torticollis, vertigo, ataxia

The vestibular nuclei receive afferent inputs from the vestibular labyrinth and cerebellum, which are integrated to maintain head and body position. Imbalance of vestibular tone can lead to abnormal head tilt [28]. In this case, ischemia of the right cerebellar hemisphere may have caused an acute vestibular tone imbalance, manifesting as left-leaning neck deviation. A third hypothesis considers the anatomical relationship between the PICA and the accessory nerve. It is plausible that the patient’s PICA occlusion led to a disruption in the blood supply to the cranial portion of the accessory nerve. Consequently, this ischemic insult to the accessory nerve could manifest as cranial nerve XI palsy, thereby resulting in the clinical finding of torticollis. While this mechanism may not be the most common cause of torticollis, it is essential to consider it as an alternative explanation for this patient’s presentation of neck tilt in the context of a PICA occlusion [13]. Most post-stroke dyskinesias, as in our case, are self-limiting, though pharmacotherapy can enable effective symptom control [10].

## Conclusions

CD is an unusual and often overlooked manifestation of PICA infarction. This case illustrates the importance of considering central causes, including cerebellar stroke, in patients presenting with acute-onset torticollis or dystonia, particularly when accompanied by vestibular or cerebellar symptoms. The interplay between cerebellar, vestibular, basal ganglia, and motor pathways may underlie such focal motor phenomena. Early neuroimaging and recognition of atypical stroke presentations can help prevent diagnostic delays and guide appropriate management. Our patient’s spontaneous improvement further supports the concept of transient post-ischemic motor pathway dysfunction rather than fixed structural damage.
